# Mediastinal paraganglioma of the aortopulmonary subtype — A surgical challenge

**DOI:** 10.1016/j.ijscr.2023.108448

**Published:** 2023-07-03

**Authors:** Muhammad Nabeel Safdar, Zohaa Faiz, Abdullah Khalid, Sara Iqbal, Saulat H. Fatimi

**Affiliations:** a Medical Student, Aga Khan University, Karachi, Pakistan; b Department of Cardiothoracic Surgery, Aga Khan University Hospital, Karachi, Pakistan

**Keywords:** Case report, Paraganglioma, Mediastinum, Median sternotomy, Surgical resection

## Abstract

**Introduction and importance:**

This case report describes the successful surgical management of a 30-year-old male with a non-functional carotid body tumor and a mediastinal paraganglioma occupying the aortopulmonary space. The report highlights the rarity of mediastinal paragangliomas and the challenges in their surgical management.

**Case presentation:**

The patient underwent pre-op angioembolisation of the carotid body tumor, followed by excision and left cervical lymph node dissection. A large mediastinal mass was identified and resected during surgery without needing a cardiopulmonary bypass.

The patient recovered well from surgery. Histopathology confirmed the diagnosis of clinically recurrent paraganglioma.

**Clinical discussion:**

Mediastinal paragangliomas are rare and challenging to manage due to their proximity to major vascular structures. Surgical intervention is the preferred treatment, but the risk of complications is high. In this case, the surgical approach involved resection of the mediastinal mass without cardiopulmonary bypass. This approach reduced the risk of complications associated with bypass procedures. The procedure's success underscores the importance of early diagnosis and prompt surgical intervention.

**Conclusion:**

This case report highlights the successful surgical management of a rare and clinically challenging mediastinal paraganglioma without cardiopulmonary bypass. The report underscores the importance of prompt diagnosis and surgical intervention in mediastinal paragangliomas.

## Highlights

• Paragangliomas are rare tumors that arise from neuroectodermal chromaffin paraganglionic cells outside the adrenal medulla.
• Mediastinal PGs are extremely rare, accounting for <0.3 % of all mediastinal neoplasms.
• A young male with a carotid body tumor and a mediastinal, non-functional PG underwent successful surgical resection without a cardiopulmonary bypass.
• Histopathology confirmed the diagnosis of clinically recurrent paraganglioma.

## 1 Introduction

Paragangliomas (PG) are neuroendocrine neoplasms originating from neuroectodermal chromaffin paraganglionic cells found outside the adrenal medulla and scattered at various locations in the body. These are similar to the pheochromocytomas arising from the chromaffin cells within the adrenal medulla. These slow-growing and hypervascular tumors have no predilection for gender and can affect people between the ages of 25–50 years. They are primarily benign, with malignant PGs reported at 93 cases per 400 million people [1]. Carotid body tumors represent 60–70 % of head and neck PGs, while Mediastinal PGs are infrequent, accounting for roughly 1–2 % of all PG and < 0.3 % of mediastinal neoplasms [2]. We report the case of a young male with a carotid body tumor and a mediastinal, non-functional PG occupying the critical aortopulmonary space, which was successfully resected without a cardiopulmonary bypass.

This case report has been reported in line with the SCARE criteria [7].

## 2 Case discussion

**Fig. 1:**
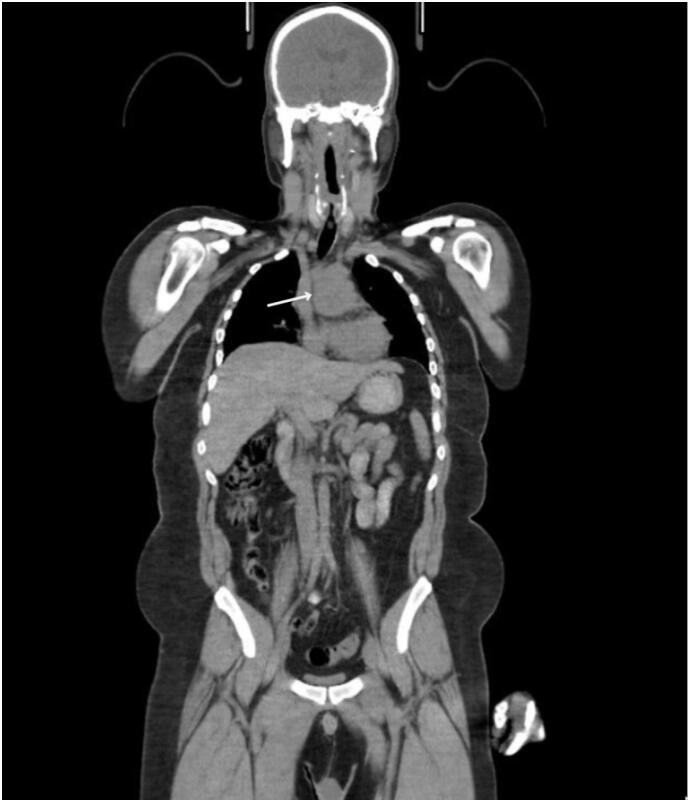
Coronal view of CT Chest showing mass occupying aortopulmonary space.

**Fig. 2:**
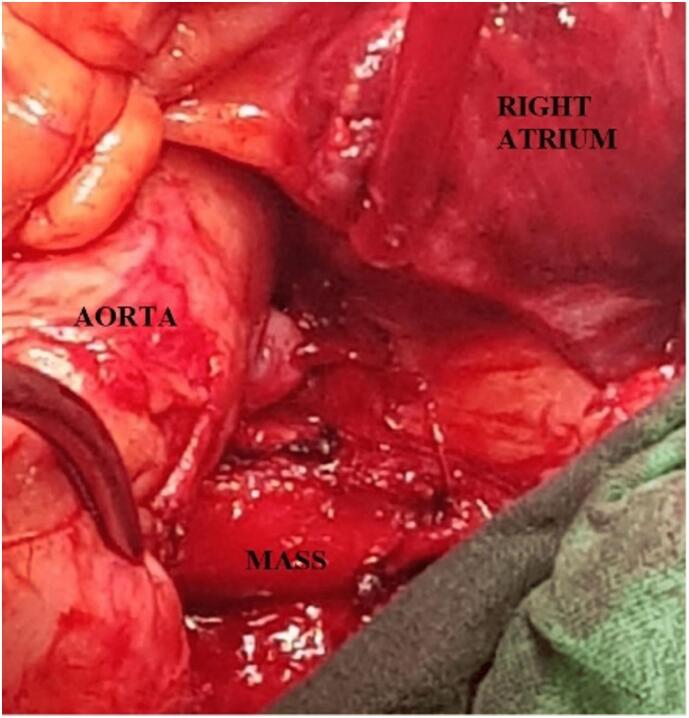
Intraoperative appearance of the mass in the right para-aortic space.

**Fig. 3:**
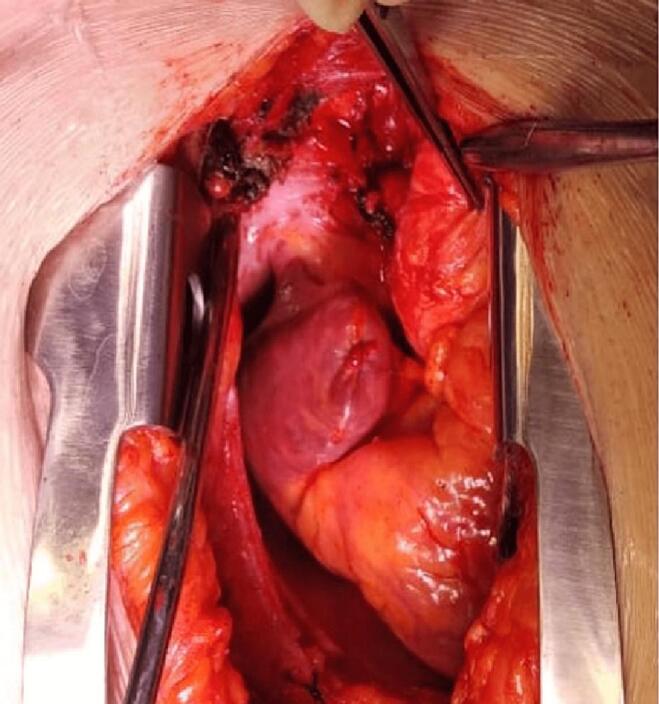
After resection of mass. Feeding vessels arising from Aorta and Right pulmonary artery can be seen ligated.

**Fig. 4:**
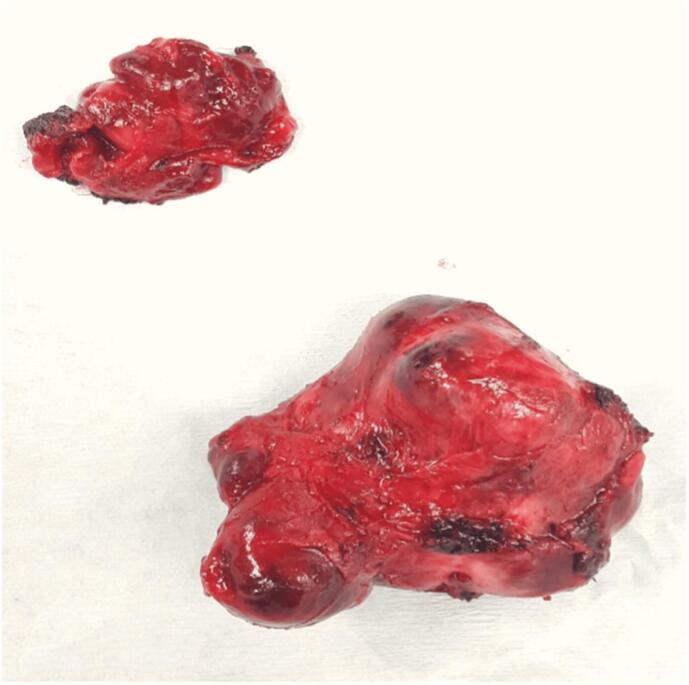
En-bloc resection of 2 masses from the Right aortopulmonary space.

A 30-year-old male with no medical or surgical history presented to our hospital six months ago with complaints of left neck swelling for the last five years. This cervical lymph node was investigated through a trucut Biopsy in his hometown that showed a Metastatic Paraganglioma (PG). He came to our hospital to seek further treatment. This gentleman was otherwise asymptomatic, and his clinical examination was unremarkable. PET-CT scan done at that point revealed a hypermetabolic soft tissue mass (3.5 × 1.7 cm) in the left parapharyngeal space and one (4.0 × 4.4 cm) in the right precarinal region along with hypermetabolic left cervical lymph nodes (Level II and III). He was diagnosed with a left carotid body (non-functional) tumor with normal urinary normetanephrine and metanephrine levels. He underwent pre-op angioembolization of his carotid body tumor that showed blood supply from branches of the left external carotid artery (ECA) embolized with PVA particles, thus, reducing 60–70 % of its blood supply. The vascular surgery department at our hospital excised the carotid body tumor and performed left cervical lymph node dissection (Fig. 1, Fig. 2, Fig. 3, Fig. 4).

Intra-operatively, a firm 5.0 × 5.0 cm mass was seen encasing the left internal carotid artery (ICA) entirely up to the base of the skull. Hence, both the left ICA and ECA were excised along with the tumor, and the ICA was reconstructed using a polytetrafluoroethylene (PTFE) vascular graft. His postoperative course was complicated by a Left Middle Cerebral Artery (MCA) territory infarct leading to right-sided hemiparesis. He made a gradual recovery from his surgery and was eventually discharged home.

Following the patient's successful surgery for the carotid body tumor and left cervical lymph node dissection, he consulted with an oncologist for further evaluation. After a thorough review of the scan by the oncologist, it was determined that no adjuvant treatment was necessary. This decision was based on several factors, including the size and location of the mediastinal mass and the absence of any signs of metastasis. The oncologist also considered the patient's medical history, including previous cancer treatments, and concluded that surgery to remove the tumor would be the most appropriate course of action. The decision to forgo adjuvant therapy was discussed with the patient, who was informed of the risks and benefits of the treatment options and agreed with the oncologist's recommendation. Six months later, the patient returned to our clinic for an assessment of his thoracic disease. The oncologist was consulted again and participated in the decision-making process for the patient's subsequent treatment plan. He remained healthy and asymptomatic throughout his postoperative period, with an 80 % recovery in his right-sided weakness on motor exam. PET-CT scan was repeated that redemonstrated a hypermetabolic mass (4.7 × 4.6 cm) in the right precarinal region, closely related to the Ascending Aorta and Superior Vena Cava (SVC) anteriorly, right pulmonary artery (PA) inferiorly and Right Main stem bronchus posteriorly. However, no residual disease was seen in the left parapharyngeal region, cervical lymph nodes, or distant metastasis. The patient was diagnosed with a Mediastinal Paraganglionoma and underwent a median sternotomy under general anesthesia for a complete resection of the mass. After pericardiotomy and excising the right peri-aortic tissue between the SVC and ascending aorta, a firm, well-encapsulated, lobulated (5.5 × 4.5 × 3.5 cm) mass was seen occupying the space behind the ascending aorta and overlying the right PA. A similar, smaller (3.5 × 2.5 × 1.5 cm) nodal mass was posterior to the central mass. These were highly vascular structures deriving their feeding and draining vessels from the aorta and Right PA, respectively, which were ligated. Considering the complex location of the mass and its proximity to major vascular structures, the Cardiopulmonary Bypass (CPB) was kept on standby. However, we performed a complete resection of the tumor of the CPB without suffering any major intraoperative hemorrhage or hemodynamic compromise. 32fr chest drains were placed in the mediastinum, and the chest was closed in a standard technique using sternal wires. The patient had an uneventful recovery postoperatively and was discharged home on the 4th Postop day. Histopathology showed tumor cells arranged in nests separated by fibrous connective tissue septae and a Zellballen pattern with faint cytoplasm and prominent nuclei. The immunohistochemical profile of tumor cells was positive for synaptophysin and S100 in the sustentacular cell, thus, confirming the diagnosis of clinically recurrent paraganglioma.

## 3 Discussion

Glenner et al. classified mediastinal PGs into two groups: 1) Aortopulmonary PG (APPG) – These originate from the parasympathetic chain in the anterior and middle mediastinum and are often present in older patients (>40 years). 2) Aorticosympathetic PG (ASPG) – these arise from the sympathetic chain in the posterior mediastinum along the costovertebral sulci and are seen in the younger population. APPGs are found in 5 locations in the thorax: [3].

1) Between ascending aorta and PA (Coronary ganglia).
2) Between ductus arteriosus and PA (Pulmonary PG).
3) Between the right Subclavian artery and the right Common carotid artery.
4) Between the left Common carotid artery and left Subclavian artery.
5) Caudal to left Subclavian artery adjacent to aortic arch [3].

Apart from the mediastinum, PG originates from the trachea, esophagus, heart, pericardium, and lungs [4]. Pathologically, APPGs do not secrete catecholamines (non-functional tumors) and mostly present with a mass effect or incidentally. On the other hand, ASPGs are similar to Pheochromocytomas since they secrete catecholamines (functional tumors) and can present with headache, facial flushing, palpitations, hyperhidrosis, dyspnea, or hypertension. To confirm the diagnosis, unfractionated metanephrines, and catecholamines can be measured in a 24-h urine sample. However, imaging is the gold standard. Typical features on CT scans include an isodense or hypodense, homogenous soft tissue density with intense enhancement. At the same time, MRI shows intermediate signal intensity on T1-weighted images and high signal intensity on T2-weighted images [5]. Local or distant metastasis on imaging is the best indicator of malignant behavior. Sites of metastasis include spread to regional lymph nodes, liver, and bones [4].

Functional and non-functional PG treatment is complete surgical resection with negative margins. Two major perioperative concerns in the case of PG include elevated risk of bleeding due to their hyper-vascular nature and proximity to major vascular structures and intra-op catecholamine crises in the case of functional subtypes. This presents with hypertension, brady- or tachycardia, and arrhythmias during surgery. These are best controlled by preoperative angiography ± embolization of functional tumors to reduce blood supply and initiate combined α and β adrenergic receptor blockade [6]. The surgical approach can be a thoracotomy for posterior mediastinal tumors or a median sternotomy for anterior and middle mediastinal neoplasms. Cardiopulmonary Bypass is used if the tumor adheres to or infiltrates a nearby major vessel or heart. There is no role of adjuvant treatment as PG are chemo- and radio-resistant; however, radiation may be required in recurrent or residual disease while chemotherapy with cyclophosphamide, vincristine, and dacarbazine is limited for non-resectable cases [1].

Prognosis after complete surgical resection is favorable, with long-term survival ranging from 62 to 84 % [5]. However, surveillance is necessary with semiannual and, afterward, annual follow-ups using imaging studies to detect recurrence and metastasis. Most PG are sporadic, but specific hereditary syndromes such as Von-Hippel-Lindau, multiple endocrine neoplasms, and neurofibromatosis increase genetic predisposition to PG [1]. Hence, genetic testing should be offered to first-degree relatives as the tumor is hereditary in 25–50 % of cases [5].

## 4 Conclusions

In conclusion, mediastinal paragangliomas are uncommon neuroendocrine tumors with the potential for malignancy. Because of their proximity to major arterial structures, they pose a surgical challenge. Resection of these tumors needs meticulous planning and execution, with cardiopulmonary bypass used only in specific circumstances. This case report describes the effective surgical treatment of an aortopulmonary mediastinal paraganglioma without needing a cardiopulmonary bypass. The scenario also emphasizes the significance of a comprehensive approach to tumor management.

## CRediT authorship contribution statement

Case report conception, design: Saulat Fatimi.

Data collection: Zohaa Faiz, Nabeel Safdar.

Manuscript writing (First draft): Zohaa Faiz, Nabeel Safdar, Abdullah Khalid.

Editing and critical review of the manuscript: Saulat Fatimi, Sara Iqbal, Abdullah Khalid.

## Guarantor

Dr. Saulat H. Fatimi.

## Funding

None.

## Ethics approval and consent to participate

The study was approved by the Institutional Review Board at Aga Khan University.

## Consent

Written informed consent was obtained from the patient for publication of this case report and accompanying images. A copy of the written consent is available for review by the Editor-in-Chief of this journal on request.

## Declaration of competing interest

None.

## Data Availability

Data can be provided upon reasonable request.
